# Inhibitory effects of sulfenimides on human and bovine carbonic anhydrase enzymes

**DOI:** 10.1080/14756366.2023.2194573

**Published:** 2023-03-27

**Authors:** Hasan Yakan, Gürkan Bilir, Şükriye Çakmak, Ömer Taş, Nalan Türköz Karakullukçu, Ercan Soydan, Halil Kütük, Coşkun Güçlü, Murat Şentürk, Tayfun Arslan, Seyhan Öztürk, Ercüment Aksakal, Deniz Ekinci

**Affiliations:** a Faculty of Education, Department of Science and Mathematics Education, Ondokuz Mayis University, Samsun, Türkiye; b Faculty of Agriculture, Department of Agricultural Biotechnology, Ondokuz Mayıs University, Samsun, Türkiye; c Vocational School, Environmental Health Programme, Sinop University, Sinop, Türkiye; d Karadeniz Advanced Technology Research and Application Centre, Ondokuz Mayis University, Samsun, Türkiye; e Faculty of Arts and Sciences, Department of Chemistry, Ondokuz Mayis University, Samsun, Türkiye; f Faculty of Agriculture, Department of Agricultural Biotechnology, Eskisehir Osmangazi University, Eskisehir, Türkiye; g Department of Biochemistry, Faculty of Paharnacy, Agri Ibrahim Cecen University, Agri, Türkiye; h Department of Chemistry, Faculty of Arts and Sciences, Giresun University, Giresun, Türkiye; i Department of Agricultural Biotechnology, Division of Animal Biotechnology, Agriculture Faculty, Akdeniz University, Antalya, Türkiye

**Keywords:** Carbonic anhydrase, inhibitor, sulfenimide, phthalimide, thiophenol

## Abstract

A series of sulfenimide derivatives (1a-i) were investigated as inhibitors of human (hCA-I, hCA-II) and bovine (bCA) carbonic anhydrase enzymes. The compounds were synthesised by the reaction of substituted thiophenols with phthalimide by means of an effective, simple and eco-friendly method and the structures were confirmed by IR, ^1^H NMR, ^13^C NMR, MS and elemental analysis. All derivatives except for the methyl derivative (**1b**) exhibited effective inhibitory action at low micromolar concentrations on human isoforms, but only four derivatives (**1e**, **1f**, **1h**, **1i**) inhibited the bovine enzyme. The bromo derivative (**1f)** was found to be strongest inhibitor of all three enzymes with KI values of 0.023, 0.044 and 20.57 µM for hCA-I, hCA-II and bCA, respectively. Results of our study will make valuable contributions to carbonic anhydrase inhibition studies for further investigations since inhibitors of this enzyme are important molecules for medicinal chemistry.

## Introduction

Carbonic anhydrase (CA) enzymes catalyse the reversible hydration of carbon dioxide to bicarbonate and proton^1^. Eight evolutionarily unrelated classes of CA families (α, β, γ, δ, ζ, η, θ, and ι) have been identified so far^2^^,^^3^. CA isoforms are involved in many physiological functions and homeostasis in animals, including respiration, bone resorption and calcification, electrolyte transport in various epithelia and biosynthesis of essential biomolecules as well as metabolic waste detoxification and tumorigenicity^4–7^. The inhibitors of CA isoenzymes are being used to develop a new class of medicines for epilepsy and glaucoma, two clinically significant conditions. Therefore, new CA inhibitors should be developed as potential therapeutic medicines. A wide variety of chemical ligands have been used to inhibit the catalytic activity of CAs such as anions, phenols, bischalcones, benzenesulfonamides, phthalocyanines and uracil derivatives^5–12^.

Sulfenimides (thiophthalimides) are divalent sulphur-containing compounds linked to trivalent nitrogen. Although both sulphur and nitrogen have lone pairs of electrons, the electronegativity of the two atoms differs, leading to bond polarisation and the formation of the reactive S-N bond, which may be broken down into electrophiles and nucleophiles. Some sulfenimides have a role as sulfenyl-transfer reagents and may be used to obtain sulfonamides^13^. *N*-Alkylthio- and *N*-arylthioimides are used as sulphur-transfer reagents. They react with thiols, hydrosulphides, alkoxides, amines, arenesulfinates, active methylene compounds, enamines, and organometallic compounds to give the corresponding sulfenylated products^14^. In addition, sulfenimides have a wide range of industrial, agrochemical and medical applications. ​They have been used as accelerating vulcanisation in the rubber industry and in the agrochemical industry as insecticides and fungicides^15^^,^^16^.

Many biological conditions have been treated with a class of organic chemical compounds made up of cyclic imides, such as phthalimide derivatives. Moreover, a number of possible derivatives of these compounds have been synthesised in order to inhibit acetylcholinesterase and butyrylcholinesterase and limit the antiproliferative activity on both tumour and normal cells^17^. Previous studies have investigated the preparation and biological activity of cyclic imides containing primary sulphonamide moieties as CA inhibitors^18^^,^^19^.

As previously mentioned, inhibitors of CA enzymes are of particular interest due to their potential to be used in medicinal chemistry, and our aim in this study is to extend earlier researches to explore novel candidates. For this reason, we analysed the effects of nine sulfenimide derivatives, which has never been tested as CA inhibitors, on human CA I, II and bCA.

## Materials and methods

**Figure 1.**
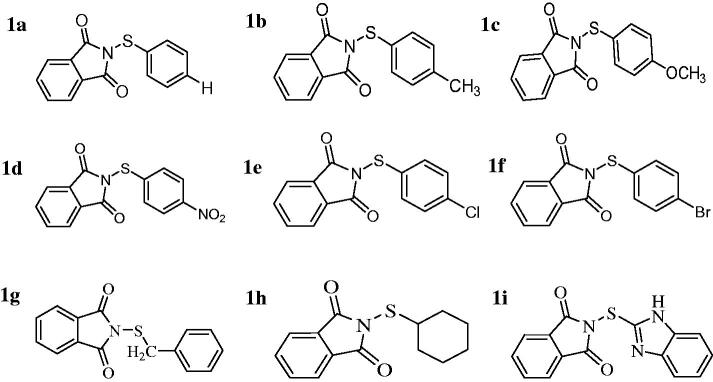
Structures of tested compounds.

Human CA enzymes were purified from erythrocytes using affinity chromatography as previously described^20^. bCA and other chemicals were obtained from Sigma-Aldrich. Synthesis and characterisation of the sulfenimides were performed as previously described^21^^,^^22^ (Figure 1).

### Hydratase activity assay

CA activity was measured by the method described by Wilbur and Anderson^23^. CO_2_–hydratase activity as an enzyme unit (EU) was calculated by using the equation (t_0_-t_c_/t_c_) where t_0_ and t_c_ are the times for pH change of the nonenzymatic and the enzymatic reactions, respectively. The inhibitory effects of the compounds **1a-i** were examined using different inhibitor concentrations. The compounds were prepared in 1 mg/ml DMSO and diluted 1000 times with distilled water. The CA activities without inhibitors were taken as 100% activity. For each inhibitor, an activity percent – [Inhibitor] graph was drawn. The IC_50_ values were obtained using the curve-fitting method and KI values were calculated from Cheng Prussof equation^24^ (Table 1).

**Table 1.** CA enzyme inhibition data with sulfenimides (KI values).

Inhibitor	KI (μM)^a^		
	hCA I	hCA II	bCA
1a	0.0585	0.0515	NA
1b	NA	NA	NA
1c	0.0379	0.060	NA
1d	0.1194	0.152	NA
1e	0.0321	0.1426	38.50
1f	**0.0230**	**0.0440**	**20.57**
1g	0.0408	0.0520	NA
1h	0.0503	0.0617	197.22
1i	0.0254	0.0546	265.96
AZA 35	0.25	0.012	40 mM

^a^Errors in the range of standart error, from 3 different assays.

## Results and discussion

Here we synthesised the sulfenimides using the simple methods described in the literature^25–29^. The pharmacokinetic properties of our compounds are shown in Table 2. As can be seen from the table, many derivatives tend to have high gastrointestinal absorption, blood brain permeability, skin permeation and inhibition of cytocrom P450 proteins^30–32^. A boiled-egg model has been also proposed for the compounds using Swissdock prediction software (Figure 5). According to the model, compounds **1d** and **1i** has the highest probability of being absorbed by the gastrointestinal tract and other derivatives might have the highest probability to permeate to the brain.

**Table 2.** Pharmacokinetic properties of the compounds obtained from SwissADME database.

Pharmacokinetic Properties	Compounds No								
	1a	1b	1c	1d	1e	1f	1g	1h	1i
Gastrointestinal absorption	High	High	High	High	High	High	High	High	High
Blood-brain barrier permeant	Yes	Yes	Yes	No	Yes	Yes	Yes	Yes	No
P-glycoprotein substrate	No	No	No	No	No	No	No	No	No
Cytochrome P450 1A2 inhibitor	Yes	Yes	Yes	No	Yes	Yes	Yes	Yes	Yes
Cytochrome P450 2C19 inhibitor	Yes	Yes	Yes	Yes	Yes	Yes	Yes	Yes	No
Cytochrome P450 2C9 inhibitor	Yes	Yes	Yes	Yes	Yes	Yes	Yes	Yes	Yes
Cytochrome P450 2D6 inhibitor	No	No	No	No	No	No	No	No	High
Cytochrome P450 3A4 inhibitor	No	No	Yes	No	No	No	No	No	No
Log Kp (skin permeation)	–6.11 cm/s	–5.94 cm/s	–6.32 cm/s	–6.51 cm/s	–5.87 cm/s	–6.10 cm/s	–6.24 cm/s	–5.97 cm/s	No

Phthalimide derivatives have been reported as transmembrane inhibitors at low nanomolar/subnanomolar concentrations of tumour-associated isoforms hCA IX and XII and less effectively inhibited cytosolic isoforms hCA I and II^33^^,^^34^. Additionally, pyridine-*N*-oxide-2-thiophenol, a thiophenol derivative, was investigated as inhibitor of CA I-XIV by Carta et al.^35^. They reported that the two mitochondrial isoforms (hCA VA – hCA VB) and isoform hCA III, were not inhibited significantly by the simple coumarin used as control but were inhibited in the low micromolar range by the thiophenol derivative. The membrane-associated isoform hCA IV was inhibited by the pyridine-*N*-oxide-2-thiophenol, with an inhibition constant of 6.13 µM. The thiophenol derivative of pyridine-*N*-oxide was a micromolar inhibitor of the tumour-associated hCA IX and hCA XII, with inhibition constants in the range of 1.72–5.40 µM. Furthermore, despite the fact that one is cytosolic (hCA XIII) and the other is transmembrane (hCA XIV), the inhibitory profiles of these two CAs with thiophenol derivative of pyridine-N-oxide were very comparable^36^. In another study by Demirdağ *et al.*, the inhibitory effects of some sulphonamides on sheep kidney CA enzyme were investigated. The IC_50_ values for the benzenesulfonamide compounds used in this study were determined to be between 1.348 and 69.31 µM^37^.

**Figure 2.**
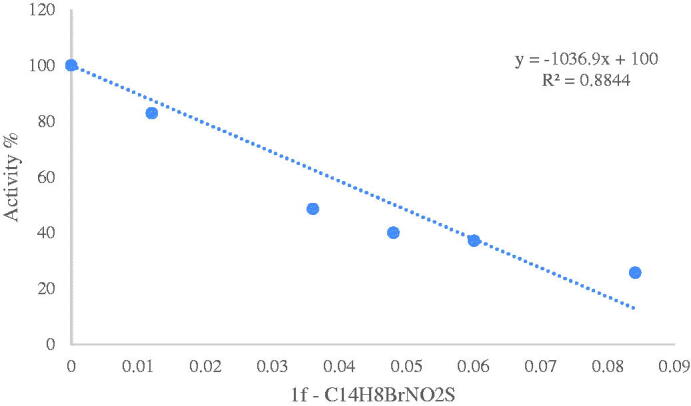
Activity %-[C_14_H_8_BrNO_2_S (µM) **(1f)**] regression analysis graph for hCA-I in the presence of five different concentrations.

**Figure 3.**
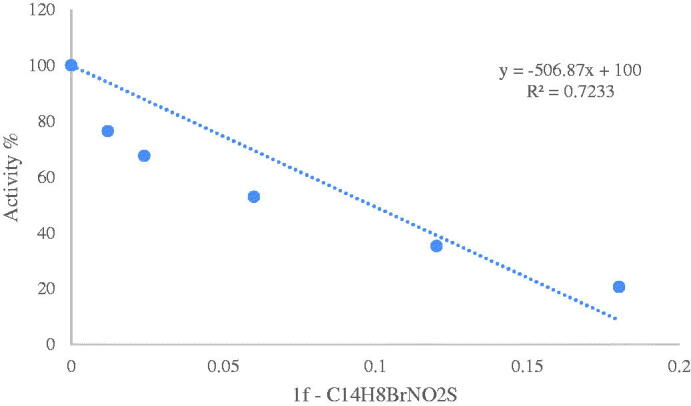
Activity %-[C_14_H_8_BrNO_2_S (µM) **(1f)**] regression analysis graph for hCA-II in the presence of five different concentrations.

**Figure 4.**
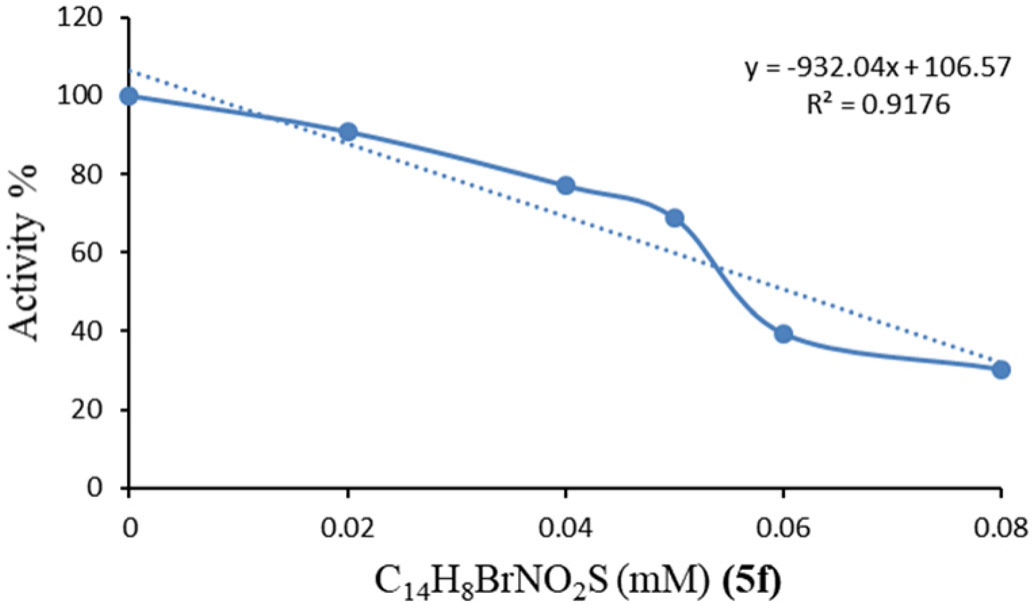
Activity %-[C_14_H_8_BrNO_2_S (µM) **(1f)**] regression analysis graph for bCA in the presence of five different concentrations.

Inhibitory effects of the obtained sulfenimides on human and bCA activities were analysed *in vitro*; IC_50_ values were calculated from the graphs and KI values were calculated using Cheng Prusoff equation. The activity% – [inhibitory] regression analysis graphs were provided for the most effective inhibitor of three enzymes (**1f**) in figures 2–4. The KI values of all compounds are shown in Table 1. Compounds **1f**, **1i, 1e, 1c, 1g, 1h, 1a** and **1d** showed quite strong inhibition on hCA-I with KI values ranging from 0.0254 to 0.1194, respectively. **1f** was found to be the most effective while **1d** exhibited the weakest action. As for hCA-II, the KI values for **1f**, **1a, 1g, 1i, 1c, 1h, 1e** and **1d** were in the range of 0.0044–0.152 µM, respectively. As seen, similar behaviour was observed for hCA-I and II isoforms such that the most effective inhibitor was **1f** and the weakest was **1d** for both two human enzymes. As clearly seen, the nitro derivative showed the weakest activity on both human isoforms. A quite much weaker inhibiton was observed for the bCA. Only four out of nine compounds showed inhibitory action on bCA with KI values ranging from 20.57 to 265.96 µM. The compound **1f** was the most effective and **1i** was the weakest inhibitor. Overall results show that compound **1f** (bromo derivative) was the most effective inhibitor for all three enzymes suggesting the efficacy of the halogen atoms.

**Figure 5.**
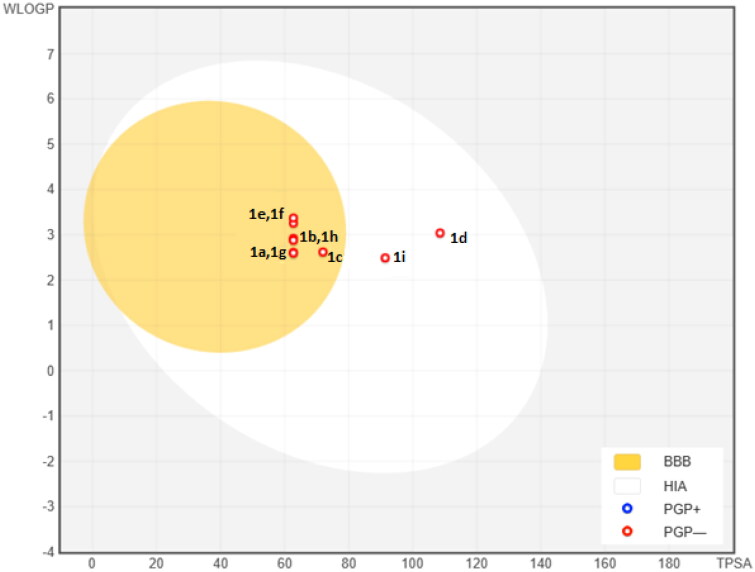
The BOILED-Egg predictive model for sulfenimide derivatives. The white region is the physicochemical space of molecules with highest probability of being absorbed by the gastrointestinal tract, and the yellow region is the physicochemical space of molecules with highest probability to permeate to the brain. Yolk and white areas are not mutually exclusive.

## Conclusion

The sulfenimides (**1a-i)** used in our study showed effective inhibition on hCA-I and -II and a rather weaker inhibition on bCA. Nevertheless, the compounds can be novel candidates of medicinal CA inhibitors due to inhibiting human isoforms I and II at nanomolar levels. The KI values of the compounds indicate that the strongest inhibitor was the bromo derivative **1f** against all three enzymes. This study confirms that sulfenimides incorporating a phthalimide-based scaffold may lead to novel CA inhibitors which could be used for medicinal purposes.
